# On randomized sketching algorithms and the Tracy–Widom law

**DOI:** 10.1007/s11222-022-10148-5

**Published:** 2023-01-19

**Authors:** Daniel Ahfock, William J. Astle, Sylvia Richardson

**Affiliations:** MRC Biostatistics Unit, University of Cambridge, Cambridge, UK

**Keywords:** Sketching, Random matrix theory, Random projection

## Abstract

There is an increasing body of work exploring the integration of random projection into algorithms for numerical linear algebra. The primary motivation is to reduce the overall computational cost of processing large datasets. A suitably chosen random projection can be used to embed the original dataset in a lower-dimensional space such that key properties of the original dataset are retained. These algorithms are often referred to as sketching algorithms, as the projected dataset can be used as a compressed representation of the full dataset. We show that random matrix theory, in particular the Tracy–Widom law, is useful for describing the operating characteristics of sketching algorithms in the tall-data regime when the sample size *n* is much greater than the number of variables *d*. Asymptotic large sample results are of particular interest as this is the regime where sketching is most useful for data compression. In particular, we develop asymptotic approximations for the success rate in generating random subspace embeddings and the convergence probability of iterative sketching algorithms. We test a number of sketching algorithms on real large high-dimensional datasets and find that the asymptotic expressions give accurate predictions of the empirical performance.

**Supplementary Information:**

The online version contains supplementary material available at 10.1007/s11222-022-10148-5.

## Introduction

Sketching is a probabilistic data compression technique that makes use of random projection (Cormode 2011; Mahoney 2011; Woodruff 2014). Suppose interest lies in a $n \times d$ dataset ${\varvec{A}}$. When *n* and or *d* are large, typical data analysis tasks will involve a heavy numerical computing load. This computational burden can be a practical obstacle for statistical learning with Big Data. When the sample size *n* is the computational bottleneck, sketching algorithms use a linear random projection to create a smaller sketched dataset of size $k \times d$, where $k \ll n$. The random projection can be represented as a $k \times n$ random matrix ${\varvec{S}}$, and the sketched dataset $\widetilde{{\varvec{A}}}$ is generated through the linear embedding $\widetilde{{\varvec{A}}}={\varvec{S}}{\varvec{A}}$. The smaller sketched dataset $\widetilde{{\varvec{A}}}$ is used as a surrogate for the full dataset ${\varvec{A}}$ within numerical routines. Through a judicious choice of the distribution on the random sketching matrix ${\varvec{S}}$, it is often possible to bound the error that is introduced stochastically into calculations given the use of the randomized approximation $\widetilde{{\varvec{A}}}$ in place of ${\varvec{A}}$

The selected distribution of the random sketching matrix ${\varvec{S}}$ can be divided into two categories, data-oblivious sketches, where the distribution is not a function of the source data ${\varvec{A}}$, and data-aware sketches, where the distribution is a function of ${\varvec{A}}$. There are also hybrid approaches where a sketching matrix ${\varvec{S}}$ is formed by taking ${\varvec{S}}=\tilde{{\varvec{S}}}{\varvec{A}}^{\textsf{T}}$ for some data-oblivious sketch $\tilde{{\varvec{S}}}$. The majority of data-aware sketches perform weighted sampling with replacement, and are closely connected to finite population survey sampling methods (Ma et al. 2015; Quiroz et al. 2018). The analysis of data-oblivious sketches requires different methods to data-aware sketches, as there are no clear ties to finite-population subsampling. In general, data-oblivious sketches generate a dataset of *k* pseudo-observations, where each instance in the compressed representation $\widetilde{{\varvec{A}}}$ has no exact counterpart in the original source dataset ${\varvec{A}}$.

Three important data-oblivious sketches are the Gaussian sketch, the Hadamard sketch and the Clarkson–Woodruff sketch. The Gaussian sketch is the simplest of these, where each element in the $k \times n$ matrix ${\varvec{S}}$ is an independent sample from a *N*(0, 1/*k*) distribution. The Hadamard sketch uses structured elements for fast matrix multiplication, and the Clarkson–Woodruff uses sparsity in ${\varvec{S}}$ for efficient computation of the sketched dataset. Other sketches that make use of sparsity include the OSNAP (Nelson and Nguyên 2013) and LESS embeddings (Derezinski et al. 2021). The comparative performance between distributions on ${\varvec{S}}$ is of interest, as there is a trade-off between the computational cost of calculating $\widetilde{{\varvec{A}}}$ and the fidelity of the approximation $\widetilde{{\varvec{A}}}$ with respect to original ${\varvec{A}}$ when choosing the type of sketch. Our results help to establish guidelines for selecting the sketching distribution.

Sketching algorithms are typically framed using stochastic $(\delta , \epsilon )$ error bounds, where the algorithm is shown to attain $(1 \pm \epsilon )$ accuracy with probability at least $1-\delta$ (Woodruff 2014). These notions are made more precise in Sect. 2. Existing bounds are typically developed from a worst-case non-asymptotic viewpoint (Mahoney 2011; Woodruff 2014; Tropp 2011). We take a different approach, and use random matrix theory to develop asymptotic approximations to the success probability given the sketching distortion factor $\epsilon$. Recent work has demonstrated the usefulness of random matrix theory to characterize the convergence rate of sketching-based iterative optimisation algorithms (Lacotte et al. 2020; Lacotte and Pilanci 2020).

Our main result is an asymptotic expression for the probability that a Gaussian based sketching algorithm satisfies general $(1 \pm \epsilon )$ probabilistic error bounds in terms of the Tracy–Widom law (Theorem 1), which describes the distribution of the extreme eigenvalues of large random matrices (Tracy and Widom 1994; Johnstone 2001). We then identify regularity conditions where other data-oblivious projections are expected to demonstrate the same limiting behavior (Theorem 3). If the motivation for using a sketching algorithm is data compression due to large *n*, the asymptotic approximations are of particular interest as they become more accurate as the computational benefits afforded by the use of a sketching algorithm increase in tandem. Empirical work has found that the quality of results can be consistent across the choice of random projections (Venkatasubramanian and Wang 2011; Le et al. 2013; Dahiya et al. 2018), and our results shed some light on this issue. An application is to determine the convergence probability when sketching is used in iterative least-squares optimisation. We test the asymptotic theory and find good agreement on datasets with large sample sizes $n \gg d$. Our theoretical and empirical results show that random matrix theory has an important role in the analysis of data-oblivious sketching algorithms for data compression.

## Sketching

### Data-oblivious sketches

As mentioned, a key component in a sketching algorithm is the distribution on ${\varvec{S}}$.

- The uniform sketch, which implements subsampling uniformly with replacement followed by a rescaling step. The Uniform projection can be represented as ${\varvec{S}}=\sqrt{n/k}\varPhi$. The random matrix $\varPhi$ subsamples *k* rows of ${\varvec{A}}$ with replacement. Element $\varPhi _{r,i}=1$ if observation *i* in the source dataset is selected in the *r*th subsampling round $(r=1, \ldots , k; \ i=1\ldots , n)$. The uniform sketch can be implemented in *O*(*k*) time.
- The Gaussian sketch, which is formed by independently sampling each element of ${\varvec{S}}$ from a *N*(0, 1/*k*) distribution. Computation of the sketched data is *O*(*ndk*).
- The Hadamard sketch is a structured random matrix (Ailon and Chazelle 2009). The sketching matrix is formed as ${\varvec{S}} = \varPhi {\varvec{H}}{\varvec{D}}/\sqrt{k}$, where $\varPhi$ is a $k \times n$ matrix and ${\varvec{H}}$ and ${\varvec{D}}$ are both $n \times n$ matrices. The fixed matrix ${\varvec{H}}$ is a Hadamard matrix of order *n*. A Hadamard matrix is a square matrix with elements that are either $+1$ or $-1$ and orthogonal rows. Hadamard matrices do not exist for all integers *n*, the source dataset can be padded with zeroes so that a conformable Hadamard matrix is available. The random matrix ${\varvec{D}}$ is a diagonal matrix where each of the *n* diagonal entries is an independent Rademacher random variable. The random matrix $\varPhi$ subsamples *k* rows of ${\varvec{H}}$ with replacement. The structure of the Hadamard sketch allows for fast matrix multiplication, reducing the complexity of the calculation of the sketched dataset relative to the Gaussian sketch, to $O(nd \log k)$ operations.
- The Clarkson–Woodruff sketch is a sparse random matrix (Clarkson and Woodruff 2013). The projection can be represented as the product of two independent random matrices, ${\varvec{S}} = {\varvec{\varGamma }}{\varvec{D}}$, where ${\varvec{\varGamma }}$ is a random $k \times n$ matrix and ${\varvec{D}}$ is a random $n \times n$ matrix. The matrix ${\varvec{\varGamma }}$ is initialized as a matrix of zeros. In each column, independently, one entry is selected and set to $+1$. The matrix ${\varvec{D}}$ is a diagonal matrix where each of the *n* diagonal entries is an independent Rademacher random variable. This results in a sparse ${\varvec{S}}$, where there is only one nonzero entry per column. The sparsity of the Clarkson–Woodruff sketch speeds up matrix multiplication, dropping the complexity of generating the sketched dataset to *O*(*nd*).

The Gaussian sketch was central to early work on sketching algorithms (Sarlos 2006). The drawback of the Gaussian sketch is that computation of the sketched data is quite demanding, taking *O*(*ndk*) operations. As such, there has been work on designing more computationally efficient random projections.

Sketch quality is commonly measured using $\epsilon$-subspace embeddings (Woodruff (2014, Chapter 2), Meng and Mahoney 2013, Yang et al. 2015). These are defined below.

#### Definition 1

$$\epsilon $$ **-subspace embedding**

For a given $n \times d$ matrix ${\varvec{A}}$, we call a $k \times n$ matrix ${\varvec{S}}$ an $\epsilon$-subspace embedding for ${\varvec{A}}$, if for all vectors ${\varvec{z}} \in \mathbb {R}^{d}$

$$\begin{aligned} (1-\epsilon )|| {\varvec{A}}{\varvec{z}}||_{2}^{2} \le ||{\varvec{S}} {\varvec{A}}{\varvec{z}}||_{2}^{2} \le (1+\epsilon )|| {\varvec{A}}{\varvec{z}}||_{2}^{2}. \end{aligned}$$

An $\epsilon$-subspace preserves the linear structure of the original dataset up to a multiplicative $(1 \pm \epsilon )$ factor. Broadly speaking, the covariance matrix of the sketched dataset $\widetilde{{\varvec{A}}}={\varvec{S}}{\varvec{A}}$ is similar to the covariance matrix of the source dataset ${\varvec{A}}$ if $\epsilon$ is small. Mathematical arguments show that the sketched dataset is a good surrogate for many linear statistical methods if the sketching matrix ${\varvec{S}}$ is an $\epsilon$-subspace embedding for the original dataset, with $\epsilon$ sufficiently small (Woodruff 2014). Suitable ranges for $\epsilon$ depend on the task of interest and structural properties of the source dataset (Mahoney and Drineas 2016).

The Gaussian, Hadamard and Clarkson–Woodruff projections are popular data-oblivious projections as it is possible to argue that they produce $\epsilon$-subspace embeddings with high probability for an arbitrary data matrix ${\varvec{A}}$. It is considerably more difficult to establish universal worst case bounds for the uniform projection (Drineas et al. 2006; Ma et al. 2015). We include the uniform projection in our discussion as it is a useful baseline. Results for sub-Gaussian sketches (Nelson and Nguyên 2013) and LESS embeddings (Derezinski et al. 2021) are also included for comparison. Table 1 summarises some key properties of different sketching matrices.

**Table 1:** Properties of different sketching matrices (see Woodruff 2014 and Derezinski et al. 2021 and the references therein)

Sketch	Sketching time	Required sketch size *k*
Gaussian	*O*(*ndk*)	$O((d+\log (1/\delta ))/\epsilon ^2)$
Hadamard	$O(nd \log k)$	$O((\sqrt{d}+\sqrt{\log n})^2(\log (d/\delta ))/ \epsilon ^2)$
Clarkson–Woodruff	*O*(*nd*)	$O(d^2/(\delta \epsilon ^2))$
Uniform	*O*(*k*)	−
Sub-Gaussian	*O*(*ndk*)	$O((d+\log (1/\delta ))/\epsilon ^2)$
LESS	$O(nd \log n + kd^2)$	$O((d \log (d/\delta ))/\epsilon ^2)$

The third column refers to the necessary sketch size *k* to obtain an $\epsilon$-subspace embedding for an arbitrary $n \times d$ source dataset with at least probability $(1-\delta )$

### Sketching algorithms

Sketching algorithms have been proposed for key linear statistical methods such as low rank matrix approximation, principal components analysis, linear discriminant analysis and ordinary least squares regression (Mahoney 2011; Woodruff 2014; Erichson et al. 2016; Falcone et al. 2021). Sketching has also been investigated for Bayesian posterior approximation (Bardenet and Maillard 2015; Geppert et al. 2017). A common thread throughout these works is the reliance on the generation of an $\epsilon$-subspace embedding. In general, $\epsilon$ serves an approximation tolerance parameter, with smaller $\epsilon$ guaranteeing higher fidelity to exact calculation with respect to some divergence measure.

An example application of sketching is ordinary least squares regression (Sarlos 2006). The sketched responses and predictors are defined as $\widetilde{{\varvec{y}}}={\varvec{S}}{\varvec{y}}, \widetilde{{\varvec{X}}}={\varvec{S}}{\varvec{X}}$. Let ${\varvec{{\varvec{\beta }}}}_{F} = {{\,\textrm{argmin}\,}}_{{\varvec{\beta }}}\Vert {\varvec{y}}-{\varvec{X}}{\varvec{\beta }} \Vert _{2}^{2}, {\varvec{\beta }}_{S} = {{\,\textrm{argmin}\,}}_{{\varvec{\beta }}}\Vert \widetilde{{\varvec{y}}}-\widetilde{{\varvec{X}}}{\varvec{\beta }} \Vert _{2}^{2}$, and $RSS_{F}=\Vert {\varvec{y}}-{\varvec{X}}{\varvec{\beta }}_{F}\Vert _{2}^{2}$. It is possible to establish the concrete bounds, that if ${{\varvec{S}}}$ is an $\epsilon$-subspace embedding for ${\varvec{A}}=({\varvec{y}}, {{\varvec{X}}})$ (Sarlos 2006), then

$$\begin{aligned} \Vert {\varvec{\beta }}_{S}- {\varvec{\beta }}_{F}\Vert _{2}^{2}&\le \dfrac{\epsilon ^2}{\sigma _{\text {min}}^2({\varvec{X}})}RSS_{F}, \end{aligned}$$

where $\sigma _{\text {min}}({\varvec{X}})$ represents the smallest singular value of the design matrix ${\varvec{X}}$. If $\epsilon$ is very small, then ${\varvec{\beta }}_{S}$ is a good approximation to ${\varvec{\beta }}_{F}$.

Given the central role of $\epsilon$-subspace embeddings (Definition 1), the success probability,

1 $$\begin{aligned} \Pr ({\varvec{S}} \text { is an}\, \epsilon \text {-subspace embedding for}\, {\varvec{A}}) \end{aligned}$$

is thus an important descriptive measure of the uncertainty attached to the randomized algorithm. The probability statement is over the random sketching matrix ${\varvec{S}}$ with the dataset ${\varvec{A}}$ treated as fixed. The embedding probability is difficult to characterize precisely using existing theory (Venkatasubramanian and Wang 2011). The bounds in Table 1 only give qualitative guidance about the embedding probability. Users will benefit from more prescriptive results in order to choose the sketch size *k*, and the type of sketch for applications (Grellmann et al. 2016; Geppert et al. 2017; Ahfock et al. 2020; Falcone et al. 2021).

Another use for sketching is in iterative solvers for ordinary least squares regression. A sketch $\widetilde{{\varvec{X}}} = {\varvec{S}}{\varvec{X}}$ can be used to generate a random preconditioner, $(\widetilde{{\varvec{X}}}^{\textsf{T}}\widetilde{{\varvec{X}}})^{-1}$, that is then applied to the normal equations ${\varvec{X}}^{\textsf{T}}{\varvec{X}}{\varvec{\beta }}={\varvec{X}}^{\textsf{T}}{\varvec{y}}$. The approach with a single sketched preconditioner is analysed in Pilanci and Wainwright (2016) and referred to as a Hessian sketch. Given some initial value ${\varvec{\beta }}^{(0)}$, the iteration is defined as

2 $$\begin{aligned} {\varvec{\beta }}^{(t+1)}&= {\varvec{\beta }}^{(t)} + (\widetilde{{\varvec{X}}}^{\textsf{T}}\widetilde{{\varvec{X}}})^{-1}{\varvec{X}}^{\textsf{T}}({\varvec{y}}-{\varvec{X}}{\varvec{\beta }}^{(t)}). \end{aligned}$$

If $\widetilde{{\varvec{X}}}^{\textsf{T}}\widetilde{{\varvec{X}}}={\varvec{X}}^{\textsf{T}}{\varvec{X}}$ the iteration will converge in a single step. The degree of noise in the preconditioner will be influenced by the sketch size *k*. A sufficient condition for convergence of the iteration (2) is that ${\varvec{S}}$ is an $\epsilon$-subspace embedding for ${\varvec{X}}$ with $\epsilon < 0.5$ (Pilanci and Wainwright 2016). As is typical with randomized algorithms, we accept some failure probability in order to relax the computational demands. It is of interest to develop expressions for the failure probability of the algorithm as a function of the sketch size *k*, as this can give useful guidelines in practice. It is possible to establish worst case bounds using the results in Table 1, however we will aim to give a point estimate of the probability. Although it is possible to improve on the iteration (2) using acceleration methods (Meng et al. 2014; Dahiya et al. 2018; Lacotte et al. 2020), we focus on the basic iteration to introduce our asymptotic techniques.

### Operating characteristics

Let the singular value decomposition of the source dataset be given by ${\varvec{A}}={\varvec{U}}{\varvec{D}}{\varvec{V}}^{\textsf{T}}$. Let $\sigma _{\text {min}}({\varvec{M}})$ and $\sigma _{\text {max}}({\varvec{M}})$ denote the minimum and maximum singular values respectively, of a matrix ${\varvec{M}}$. Likewise, let $\lambda _{\text {min}}({\varvec{M}})$ and $\lambda _{\text {max}}({\varvec{M}})$ denote the minimum and maximum eigenvalues of a matrix ${\varvec{M}}$. It is possible to show

3 $$\begin{aligned}&\Pr ({\varvec{S}} \text { is an}\, \epsilon \text {-subspace embedding for}\, {\varvec{A}}) \nonumber \\&\quad = \Pr (\sigma _{\text {max}}({\varvec{I}}_{d}-{\varvec{U}}^{\textsf{T}}{\varvec{S}}^{\textsf{T}}{\varvec{S}}{\varvec{U}}) \le \epsilon ), \end{aligned}$$

where ${\varvec{U}}$ is the $n \times d$ matrix of left singular vectors of the source data matrix ${\varvec{A}}$ (Woodruff 2014). Now as

4 $$\begin{aligned} \sigma _{\text {max}}({\varvec{I}}_{d}-{\varvec{U}}^{\textsf{T}}{\varvec{S}}^{\textsf{T}}{\varvec{S}}{\varvec{U}})&= \text {max}(\vert \lambda _{\text {max}}({\varvec{I}}_{d}-{\varvec{U}}^{\textsf{T}}{\varvec{S}}^{\textsf{T}}{\varvec{S}}{\varvec{U}}) \vert , \nonumber \\&\quad \vert \lambda _{\text {min}}({\varvec{I}}_{d}-{\varvec{U}}^{\textsf{T}}{\varvec{S}}^{\textsf{T}}{\varvec{S}}{\varvec{U}}) \vert ) \nonumber \\&= \text {max}(|1-\lambda _{\text {min}}({\varvec{U}}^{\textsf{T}}{\varvec{S}}^{\textsf{T}}{\varvec{S}}{\varvec{U}}) |,\nonumber \\&\quad |1-\lambda _{\text {max}}({\varvec{U}}^{\textsf{T}}{\varvec{S}}^{\textsf{T}}{\varvec{S}}{\varvec{U}}) |) , \end{aligned}$$

the extreme eigenvalues of ${\varvec{U}}^{\textsf{T}}{\varvec{S}}^{\textsf{T}}{\varvec{S}}{\varvec{U}}$ are the critical factor in generating $\epsilon$-subspace embeddings. The convergence behavior of the basic iteration (2) is also tied to the eigenvalues of ${\varvec{U}}^{\textsf{T}}{\varvec{S}}^{\textsf{T}}{\varvec{S}}{\varvec{U}}$ where ${\varvec{A}}={\varvec{X}}$. Providing that $(\widetilde{{\varvec{X}}}^{\textsf{T}}\widetilde{{\varvec{X}}})$ is of rank *d*, the maximum eigenvalue satisfies

$$\begin{aligned} \lambda _{\text {max}}((\widetilde{{\varvec{X}}}^{\textsf{T}}\widetilde{{\varvec{X}}})^{-1}{\varvec{X}}^{\textsf{T}}{\varvec{X}})&= \lambda _{\text {max}}(({\varvec{U}}^{\textsf{T}}{\varvec{S}}^{\textsf{T}}{\varvec{S}}{\varvec{U}})^{-1}). \end{aligned}$$

From standard results on iterative solvers (Hageman and Young 2012), a necessary and sufficient condition for the iteration to converge is $\underset{t \rightarrow \infty }{\lim }\Vert {\varvec{\beta }}_{F} - {\varvec{\beta }}^{(t)} \Vert _{2} = 0$ if and only if $\lambda _{\text {max}}((\widetilde{{\varvec{X}}}^{\textsf{T}}\widetilde{{\varvec{X}}})^{-1}{\varvec{X}}^{\textsf{T}}{\varvec{X}}) < 2$. The probability of convergence can then be expressed as

5 $$\begin{aligned} \Pr \left( \underset{t \rightarrow \infty }{\lim }\Vert {\varvec{\beta }}_{F} - {\varvec{\beta }}^{(t)} \Vert _{2} = 0\right)&= \Pr (\lambda _{\text {min}}({\varvec{U}}^{\textsf{T}}{\varvec{S}}^{\textsf{T}}{\varvec{S}}{\varvec{U}}) > 0.5). \end{aligned}$$

Most existing results on the probabilities (3) and (5) are finite sample lower bounds (Tropp 2011; Nelson and Nguyên 2013; Meng 2014). Worst case bounds can be conservative in practice, and there is value in developing other methods to characterize the performance of randomized algorithms (Halko et al. 2011; Raskutti and Mahoney 2014; Lopes et al. 2018; Dobriban and Liu 2018). The embedding probability (3) and the convergence probability (5) are related to the extreme eigenvalues of ${\varvec{U}}^{\textsf{T}}{\varvec{S}}^{\textsf{T}}{\varvec{S}}{\varvec{U}}$. In Sect. 3 we study this distribution for the Gaussian sketch and develop a Tracy–Widom approximation. The approximation is then extended to the Clarkson–Woodruff and Hadamard sketches in Sect. 4.

## Gaussian sketch

### Exact representations

Meng (2014, Sect. 2.3) notes that when using a Gaussian sketch, it is instructive to consider directly the distribution of the random variable $\sigma _{\text {max}}({\varvec{I}}_{d}-{\varvec{U}}^{\textsf{T}}{\varvec{S}}^{\textsf{T}}{\varvec{S}}{\varvec{U}})$ to study the embedding probability (3). Consider an arbitrary $n \times d$ data matrix ${\varvec{A}}$. As ${\varvec{S}}$ is a matrix of independent Gaussians with mean zero and variance 1/*k*, ${\varvec{S}}{\varvec{U}}$ is a $k \times d$ matrix of where each row has a $N(0, {\varvec{I}}_{d}/k)$ distribution. It follows from the definition of a Wishart distribution that

$$\begin{aligned} {\varvec{U}}^{\textsf{T}}{\varvec{S}}^{\textsf{T}}{\varvec{S}}{\varvec{U}}&\sim \text {Wishart}\left( k, {\varvec{I}}_{d}/k\right) . \end{aligned}$$

The key term ${\varvec{U}}^{\textsf{T}}{\varvec{S}}^{\textsf{T}}{\varvec{S}}{\varvec{U}}$ is in some sense a pivotal quantity, as its distribution is invariant to the actual values of the data matrix ${\varvec{A}}$. When using a Gaussian sketch, the probability of obtaining an $\epsilon$-subspace embedding has no dependence on the number of original observations *n*, or on the values in the data matrix ${\varvec{A}}$. This is a useful property for a data-oblivious sketch, as it is possible to develop universal performance guarantees that will hold for any possible source dataset. This invariance property is also noted in Meng (2014), although the derivation is different.

Let us define the random matrix ${\varvec{W}} \sim \text {Wishart}(k, {\varvec{I}}_{d}/k)$. The success probability of interest can then be expressed in terms of the extreme eigenvalues of the Wishart distribution The embedding probability of interest has the representation:

6 $$\begin{aligned}&\Pr ({\varvec{S}} \text { is an}\, \epsilon \text {-subspace embedding for}\, {\varvec{A}})\nonumber \\&\quad = \Pr \left( | 1- \lambda _{\text {min}}({\varvec{W}})| \le \epsilon , | 1- \lambda _{\text {max}}({\varvec{W}})| \le \epsilon \right) . \end{aligned}$$

where we have made use of the expression for the maximum singular value (4).

It is difficult to obtain a mathematically tractable expression for the embedding probability as it involves the joint distribution of the extreme eigenvalues (Chiani 2017). Meng forms a lower bound on the probability (6) using concentration results on the eigenvalues of the Wishart distribution.

The convergence probability (5), can also be related to the eigenvalues of the Wishart distribution. Assuming $k \ge d$, the matrix $\widetilde{{\varvec{X}}}^{\textsf{T}}\widetilde{{\varvec{X}}}$ has full rank with probability one. As such, using the same pivotal quantity ${\varvec{U}}^{\textsf{T}}{\varvec{S}}^{\textsf{T}}{\varvec{S}}{\varvec{U}}$ as before,

7 $$\begin{aligned} \Pr \left( \underset{t \rightarrow \infty }{\lim }\Vert {\varvec{\beta }}_{F} - {\varvec{\beta }}^{(t)} \Vert _{2} = 0\right)&= \Pr (\lambda _{\text {min}}({\varvec{W}}) > 0.5), \end{aligned}$$

where ${\varvec{W}}\sim \text {Wishart}(k, {\varvec{I}}_{d}/k)$. The convergence probability (7) has no dependence on the specific response vector ${\varvec{y}}$ or design matrix ${\varvec{X}}$ under consideration. Problem invariance is a highly desirable property for a randomized iterative solver (Roosta-Khorasani and Mahoney 2016; Lacotte et al. 2020). Both the embedding probability and the convergence probability are related to the extreme eigenvalues of the Wishart distribution. The extreme eigenvalues of Wishart random matrices are a well studied topic in random matrix theory (Edelman 1988), and we can make use of existing results to analyse the operating characteristics of sketching algorithms. In the following section we develop approximations to the embedding probability and the convergence probability in the asymptotic regime:

8 $$\begin{aligned} n,d,k \rightarrow \infty , \quad n \gg k, \quad d/k \rightarrow \alpha \in (0, 1]. \end{aligned}$$

The regime (8) can be viewed as an interesting stress test for sketching algorithms for data compression. A key feature of the bounds in Table 1 for the embedding probability is that there is either no dependence or weak dependence on the sample size *n*. Working in the regime where $n \gg k$ is natural to demonstrate the effectiveness of the sketching algorithm. Allowing the number of variables *d* to grow with *n* allows for the difficulty of the compression task to increase with *n*. Fixing the variables to sketch size ratio *d*/*k* is important to ensure that estimates derived from the sketched dataset remain stable. The benefit in adopting this regime is the ability to obtain explicit estimates for the embedding probability that are easily computable.

**Fig. 1:**
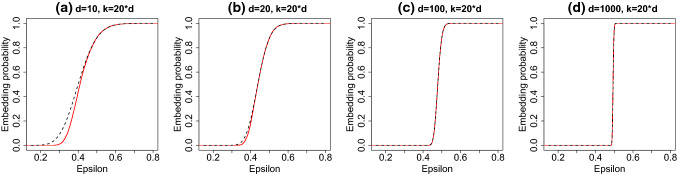
Accuracy of Tracy–Widom approximation for embedding probability (6) for the Gaussian sketch. The dashed black line gives the asymptotic limit, the solid red line gives the empirical probability. When $d\ge 20$ the approximation given in Theorem 1 is very accurate. (Color figure online)

### Random matrix theory

Random matrix theory involves the analysis of large random matrices (Bai and Silverstein 2010). The Tracy–Widom law is an important result in the study of the extreme eigenvalue statistics (Tracy and Widom 1994). Johnstone (2001) showed that Tracy–Widom law gives the asymptotic distribution of the maximum eigenvalue of a $\text {Wishart}(k, {\varvec{I}}_{d}/k)$ matrix after appropriate centering and scaling. In subsequent work Ma (2012) showed that the rate of convergence could be improved from ${O}(d^{-1/3})$ to ${O}(d^{-2/3})$ by using different centering and scaling constants than in Johnstone (2001). We build from the convergence results given by Ma.

The R package RMTstat contains a number of functions for working with the Tracy–Widom distribution (Johnstone et al. 2014). The main application of the Tracy–Widom law to statistical inference has been its use in hypothesis testing in high-dimensional statistical models (Johnstone 2006; Bai and Silverstein 2010). The Tracy–Widom law has also been demonstrated to be a universal law for the extreme eigenvalues for a wide range of random matrices beyond the Wishart (Bao et al. 2015). To the best of our knowledge, the connection to sketching algorithms has not been explored in great depth. The Tracy–Widom law can be used to approximate the embedding probability (3).

#### Theorem 1

Suppose we have an arbitrary $n \times d$ data matrix ${\varvec{A}}$ where $n >d$ and ${\varvec{A}}$ is of rank *d*. Furthermore assume we take a Gaussian sketch of size *k*. Consider the limit in *n*, *k* and *d*, such that $d/k \rightarrow \alpha$ with $\alpha \in (0, 1]$. Define centering and scaling constants $\mu _{k,d}$ and $\sigma _{k,d}$ as

$$\begin{aligned} \mu _{k,d}&= k^{-1}(\sqrt{k-1/2}+\sqrt{d-1/2})^{2}, \quad \\ \sigma _{k,d}&= \dfrac{k^{-1}(\sqrt{k-1/2}+\sqrt{d-1/2})}{\left( 1/{\sqrt{k-1/2}}+1/{\sqrt{d-1/2}}\right) ^{1/3}}. \end{aligned}$$

Set $Z \sim F_{1}$ where $F_{1}$ is the Tracy–Widom distribution. Let $\psi _{n,k,d}$ give the exact embedding probability and let $\widehat{\psi }_{n,k,d}$ give the asymptotic approximation to the embedding probability:

$$\begin{aligned} \psi _{n,k,d}&= \Pr \left( {\varvec{S}} is an \, \epsilon -subspace embedding for \, {\varvec{A}}\right) , \quad \\ \widehat{\psi }_{n,k,d}&= \Pr \left( Z \le \dfrac{\epsilon +1-\mu _{k,d}}{\sigma _{k,d}}\right) . \end{aligned}$$

Then asymptotically in *n*, *d* and *k*, for any $\epsilon >0$,

$$\begin{aligned} \underset{n,d,k \rightarrow \infty }{\lim }\ \left|\psi _{n,k,d} - \widehat{\psi }_{n,k,d} \right|&=0 \end{aligned}$$

Furthermore, for even *d*, $\left|\psi _{n,k,d}-\widehat{\psi }_{n,k,d} \right|= {O}(d^{-2/3})$.

The proof is given in the supplementary material.

The convergence probability of the iterative algorithm (5) can also be approximated using the Tracy–Widom law.

#### Theorem 2

Suppose we have an arbitrary $n \times d$ data matrix ${\varvec{A}}$ where $n >d$ and ${\varvec{A}}$ is of rank *d*. Furthermore, assume we take a Gaussian sketch of size *k*. Consider the limit in *n*, *k* and *d*, such that $d/k \rightarrow \alpha$ with $\alpha \in (0, 1]$. Set

$$\begin{aligned} \mu _{k,d}&= (\sqrt{k-1/2}-\sqrt{d-1/2})^{2},\\ \sigma _{k,d}&= (\sqrt{k-1/2}-\sqrt{d-1/2})\left( \dfrac{1}{\sqrt{k-1/2}}-\dfrac{1}{\sqrt{d-1/2}}\right) ^{1/3}, \end{aligned}$$

and define the following centering and scaling constants $\tau _{k,d} = \sigma _{k,d}/\mu _{k,d}, \nu _{k,d} = \log (\mu _{k,d})-\log k -\tau _{k,d}^2/8$. Set $Z \sim F_{1}$, where $F_{1}$ is the Tracy–Widom distribution. Let $\gamma _{n,k,d}$ give the exact convergence probability, and $\widehat{\gamma }_{n,k,d}$ give the asymptotic approximation to the convergence probability:

$$\begin{aligned} \gamma _{n,k,d}&= \Pr \left( \underset{t \rightarrow \infty }{\lim }\Vert {\varvec{\beta }}_{F} - {\varvec{\beta }}^{(t)} \Vert _{2} = 0\right) , \quad \\ \widehat{\gamma }_{n,k,d}&= \Pr \left( Z \le \dfrac{\nu _{k,d}-\log (1/2)}{\tau _{k,d}}\right) . \end{aligned}$$

Then for all starting values ${\varvec{\beta }}^{(0)}$, asymptotically in *n*, *d* and *k*,

$$\begin{aligned} \underset{n,d,k \rightarrow \infty }{\lim }\ \left|\gamma _{n,k,d}-\widehat{\gamma }_{n,k,d} \right|= 0. \end{aligned}$$

Furthermore, for even *d*, $\left|\gamma _{n,k,d}-\widehat{\gamma }_{n,k,d} \right|= {O}(d^{-2/3})$.

The proof is given in the supplementary material.

The embedding probability for the Gaussian sketch can be estimated by simulating ${\varvec{W}} \sim \text {Wishart}(k, {\varvec{I}}_{d}/k)$ and using the empirical distribution of the random variable $\sigma _{\text {max}}\left( {\varvec{I}}_{d} - {\varvec{W}}\right)$. To assess the accuracy of the approximation in Theorem 1, we generated $B=10,000$ random Wishart matrices ${\varvec{W}}^{[1]}, \ldots , {\varvec{W}}^{[B]}$. For each simulated matrix ${\varvec{W}}^{[b]}$ we computed the distortion factor $\epsilon ^{[b]} = \sigma _{\text {max}}({\varvec{I}}_{d}-{\varvec{W}}^{[b]})$ for $b=1, \ldots , B$. The simulated distortion factors $\epsilon ^{[1]}, \ldots , \epsilon ^{[B]}$ were used to give a Monte Carlo estimate of the embedding probability:

9 $$\begin{aligned} \widehat{\Pr }({\varvec{S}} \text { is an } \epsilon \text {-subspace embedding for } {\varvec{A}})&= \dfrac{1}{B}\sum _{b=1}^{B}\mathbbm {1}(\epsilon ^{[b]}\nonumber \\&\le \epsilon ). \end{aligned}$$

We used the ARPACK library (Lehoucq et al. 1998) to compute the maximum singular values $\sigma _{\text {max}}({\varvec{I}}_{d}-{\varvec{W}}^{[b]})$. The estimated embedding probabilities are displayed in Fig. 1 for different dimensions *d*. The sketch size to variables ratio, *k*/*d*, was held fixed at 20. The solid red line shows the empirical probability of obtaining an $\epsilon$-subspace embedding. The dashed black line gives the Tracy–Widom approximation given in Theorem 1. The agreement is consistently good over dimensions *d*, and the range of sketch sizes *k* that were considered.

## Computationally efficient sketches

### Asymptotics for the extreme eigenvalues

Asymptotic methods are useful to analyse data-oblivious sketches that do not admit interpretable finite sample distributions (Li et al. 2006; Ahfock et al. 2020; Lacotte et al. 2020). Here we describe the limiting behavior of the sketched algorithms for fixed *k* and *d* as the number of source observations *n* increases.

Under an assumption on the limiting leverage scores of the source data matrix, we can establish a limit theorem for the Hadamard and Clarkson–Woodruff sketches.

#### Assumption 1

Define the singular value decomposition of the $n \times d$ source dataset as ${\varvec{A}}_{(n)}={\varvec{U}}_{(n)}{\varvec{D}}_{(n)}{\varvec{V}}_{(n)}^{\textsf{T}}$. Let ${\varvec{u}}_{(n)i}^{\textsf{T}}$ give the *i*th row in ${\varvec{U}}_{(n)}$. Assume that the maximum leverage score tends to zero, that is

$$\begin{aligned} \lim _{n \rightarrow \infty } \underset{i=1, \ldots , n}{\text {max}} \Vert {\varvec{u}}_{(n)i} \Vert _{2}^{2} = 0. \end{aligned}$$

Assuming that ${\varvec{A}}_{(n)}$ is of rank *d*, the leverage scores have an important standardization property in that

10 $$\begin{aligned} \sum _{i=1}^{n}\Vert {\varvec{u}}_{(n)i} \Vert _{2}^{2}=d. \end{aligned}$$

Assumption 1 represents an asymptotic negligibility condition on the significance of any single observation. The same assumption is made in the analysis of high-dimensional regression models in Huber (1973, Proposition 2.2). Similar to the Lindeberg-Feller condition (Van Der Vaart 1998), Assumption 1 requires that the contribution of any single observation to the total variance in the source dataset (10) is arbitrarily small for sufficiently large values of *n*. Assumption 1 is expected to hold if there are no extreme outliers in the source dataset.

The asymptotic probability of obtaining an $\epsilon$-subspace embedding for the Hadamard and Clarkson–Woodruff sketches can be related to the Wishart distribution.

#### Theorem 3

Consider a sequence of arbitrary $n \times d$ data matrices ${\varvec{A}}_{(n)}$, where each data matrix is of rank *d*, and *d* is fixed. Let ${\varvec{A}}_{(n)}={\varvec{U}}_{(n)}{\varvec{D}}_{(n)}{\varvec{V}}_{(n)}^{\textsf{T}}$ represent the singular value decomposition of ${\varvec{A}}_{(n)}$. Let ${\varvec{S}}_{(n)}$ be a $k \times n$ Hadamard or Clarkson–Woodruff sketching matrix where *k* is also fixed. Suppose that Assumption 1 is satisfied. Then as *n* tends to infinity with *k* and *d* fixed,

$$\begin{aligned}&\underset{n \rightarrow \infty }{\lim }\Pr \left( {\varvec{S}}_{(n)} is an \, \epsilon -subspace embedding for \, {\varvec{A}}_{(n)}\right) \\&\quad = \Pr \left( \sigma _{max }({\varvec{I}}_{d}-{\varvec{W}}) \le \epsilon \right) , \end{aligned}$$

where $${\varvec{W}} \sim Wishart (k, {\varvec{I}}_{d}/k)$$.

The proof is given in the supplementary material.

Theorem 3 states the the embedding probability for the Hadamard and Clarkson–Woodruff sketches converges to that of the Gaussian sketch as $n \rightarrow \infty$. Therefore, Theorem 1 can also be used to approximate the embedding probability. Empirical studies have shown that the Hadamard and Clarkson–Woodruff sketches can give similar quality results to the Gaussian projection (Venkatasubramanian and Wang 2011; Le et al. 2013; Dahiya et al. 2018). Theorem 3 helps to characterize situations where this phenomenon is expected to be observed.

#### Remark 1

The same line of proof used in Theorem 3 can be used to show that the convergence probability of (2) using the Hadamard and Clarkson–Woodruff projections converges to that of the Gaussian sketch under Assumption 1. Theorem 2 also gives an asymptotic approximation for the Hadamard and Clarkson–Woodruff sketches.

It remains to establish a formal limit theorem in terms of the Tracy–Widom distribution for the Hadamard and Clarkson–Woodruff sketches. The proof of Theorem 3 treats *k* and *d* as fixed, with only *n* being taken to infinity. It is possible that Assumption 1 on the leverage scores will remain sufficient in the expanding dimension scenario. For any *d*, the maximum leverage score must be greater than the average leverage score,

$$\begin{aligned} \underset{i=1,\ldots , n}{\text {max}} \Vert {\varvec{u}}_{(n)i} \Vert _{2}^{2}&\ge \dfrac{1}{n}\sum _{i=1}^{n} \Vert {\varvec{u}}_{(n)i} \Vert _{2}^{2} = \dfrac{d}{n}. \end{aligned}$$

If we maintain that Assumption 1 holds on the leverage scores as $n,d,k \rightarrow \infty$, this implies that $d/n \rightarrow 0$. As we have assumed that our primary motivation for sketching is data compression when $n \gg d$, we feel that analysis in the asymptotic regime $d/n \rightarrow 0$ is reasonable for this use-case setting. The asymptotic approximations developed here are recommended for applications of sketching in tall-data problems where $n \gg d$.

The key result is that the Hadamard and Clarkson–Woodruff sketches behave like the Gaussian projection for large *n*, with *k* and *d* fixed. If the Tracy–Widom approximation in Theorem 1 is good for finite *k* and *d* with the Gaussian sketch, then it should hold well for the Hadamard and Clarkson–Woodruff projections for *n* sufficiently large.

### Uniform sketch

It is considerably more difficult to approximate the embedding probability for the uniform sketch compared to the other data-oblivious projections. Vershynin (2010) provides a bound for the uniform sketch that is useful for comparative purposes.

#### Theorem 4

(Vershynin (2010), Theorem 5.41) Consider an $n \times d$ matrix ${\varvec{U}}$ such that ${\varvec{U}}^{\textsf{T}}{\varvec{U}}={\varvec{I}}_{d}$. Let ${\varvec{u}}_{i}^{\textsf{T}}$ represent the *i*-th row in ${\varvec{U}}$ for $i=1, \ldots , n$. Let *r* give an upper bound on the leverage scores, so

$$\begin{aligned} \underset{i=1, \ldots , n}{\max }\ \ \Vert {\varvec{u}}_{i} \Vert _{2}^{2} \le r. \end{aligned}$$

Let ${{\varvec{S}}}$ be a uniform sketch of size *k*. Then for every $t \ge 0$, with probability at least $1-2d\exp (-ct^2)$ one has

$$\begin{aligned} 1- t\sqrt{\dfrac{rn}{k}} \le \sigma _{min }({\varvec{S}}{\varvec{U}}) \le \sigma _{max }({\varvec{S}}{\varvec{U}}) \le 1+t\sqrt{\dfrac{rn}{k}}, \end{aligned}$$

where $c > 0$ is an absolute constant.

Theorem 4 is a minor reformulation of the result presented in Vershynin (2010), this is elaborated on in the supplementary material.

Theorem 4 can be used to give a lower bound on the probability of obtaining an $\epsilon$-subspace embedding. Both Theorems 4 and 3 involve the maximum leverage score. Holding *k* and *d* fixed, in order for the bound in Theorem 4 to remain controlled as the sample size *n* increases, the maximum leverage score *r* must decrease at a sufficient rate. In contrast, Assumption 1 does not enforce a rate of decay on the maximum leverage score, only that it eventually tends to zero as $n \rightarrow \infty$. This suggests that the uniform projection could be more sensitive to the maximum leverage score than the Gaussian, Hadamard and Clarkson–Woodruff projections.

### Asymptotics for the empirical spectral distribution

An alternative approach to estimate the embedding probability is to use the limiting empirical spectral distribution of ${\varvec{M}}_{d}={\varvec{U}}^{\textsf{T}}{\varvec{S}}^{\textsf{T}}{\varvec{S}}{\varvec{U}}$. For a random Hermitian matrix ${\varvec{M}}_{d}$ of size $d \times d$, the empirical spectral distribution of ${\varvec{M}}_{d}$ is the cumulative distribution function of its eigenvalues $\lambda _{1} \le \cdots \le \lambda _{d}$, i.e, $F_{M_{d}}(x):= \tfrac{1}{d}\sum _{j=1}^{d}\mathbbm {1}(\lambda _{j} \le x)$ for $x \in \mathbb {R}$.

Lacotte et al. (2020) derive the limiting empirical spectral distribution of ${\varvec{M}}_{d}={\varvec{U}}^{\textsf{T}}{\varvec{S}}^{\textsf{T}}{\varvec{S}}{\varvec{U}}$ for the Hadamard sketch in the asymptotic regime where $\lim _{n \rightarrow \infty } {d}/{n} = \gamma \in (0, 1)$, $\lim _{n \rightarrow \infty } {k}/{n} = \xi \in (\gamma , 1)$ and $\lim _{n \rightarrow \infty } {d}/{k} = \alpha$. The extreme eigenvalues of ${\varvec{M}}_{d}={\varvec{U}}^{\textsf{T}}{\varvec{S}}^{\textsf{T}}{\varvec{S}}{\varvec{U}}$ under the Hadamard sketch converge pointwise to (Lacotte and Pilanci 2020)

11 $$\begin{aligned} \lambda _{\text {min}}({\varvec{U}}^{\textsf{T}}{\varvec{S}}^{\textsf{T}}{\varvec{S}}{\varvec{U}})&= (\sqrt{1-\gamma } - \sqrt{(1-\xi )\alpha })^2. \end{aligned}$$

12 $$\begin{aligned} \lambda _{\text {max}}({\varvec{U}}^{\textsf{T}}{\varvec{S}}^{\textsf{T}}{\varvec{S}}{\varvec{U}})&= (\sqrt{1-\gamma } + \sqrt{(1-\xi )\alpha })^2. \end{aligned}$$

The results (11) and (12) imply the convergence result for the maximum singular value

$$\begin{aligned} \sigma _{\text {max}}({\varvec{I}}_{d}-{\varvec{U}}^{\textsf{T}}{\varvec{S}}^{\textsf{T}}{\varvec{S}}{\varvec{U}})&= \text {max}(|1-(\sqrt{1-\gamma } - \sqrt{(1-\xi )\alpha })^2 |, \\&\quad |1-(\sqrt{1-\gamma } + \sqrt{(1-\xi )\alpha })^2 |). \end{aligned}$$

The resulting approximation to the embedding probability is then

13 $$\begin{aligned} \Pr ({\varvec{S}} \text { is an}\, \epsilon \text {-subspace embedding})&= {\left\{ \begin{array}{ll} 1 \text{ if } \epsilon \ge \sigma ^{*} \\ 0 \text{ if } \epsilon < \sigma ^{*}, \end{array}\right. } \end{aligned}$$

where $$\sigma ^{*}=\text {max}(|1-(\sqrt{1-\gamma } - \sqrt{(1-\xi )\alpha })^2 |, |1-(\sqrt{1-\gamma } + \sqrt{(1-\xi )\alpha })^2 |)$$.

**Fig. 2:**
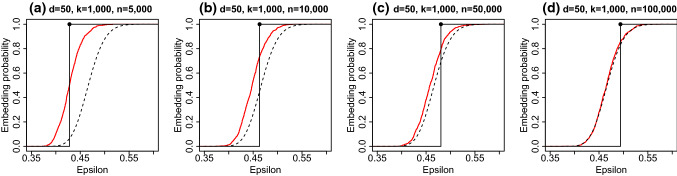
Embedding probability for the Hadamard sketch. The solid red line gives the empirical probability. The dashed black line gives the Tracy–Widom approximation to the embedding probability. The step function represents the approximation to the embedding probability using the limiting empirical spectral distribution. (Color figure online) (13)

We estimated the embedding probability using the Hadamard sketch on simulated data with $d=50, k=1000$ and $n \in \{5000,10000,50000,100000 \}$ over 1000 sketches. Each row in the source dataset was an independent draw from a $N(0, \varSigma )$ distribution where $\varSigma _{ij}=\rho ^{|i-j|}$ and $\rho =0.5$. Lacotte et al. (2020) consider a slight variant of the Hadamard sketch where the subsampling matrix $\varPhi$ is constructed using subsampling without replacement. In the simulation, the Hadamard sketch was implemented using subsampling without replacement. Figure 2 compares the empirical embedding probability (solid red line) to the to the Tracy–Widom approximation in Theorem 1 (black dashed line) and the empirical spectral distribution approximation in (13) (step function).

When *d*/*n* is large, the approximation to the embedding probability (13) suggests that the Hadamard sketch will perform better than is predicted by the Tracy–Widom law. In panel (a) of Figure 2 where $d=50, n=5000$, the empirical embedding probability for the Hadamard sketch is shifted to the left compared to the Tracy–Widom limit, which indicates superior performance. As *n* increases and $d/n \rightarrow 0$, the Tracy–Widom approximation becomes more accurate as predicted by Theorem 3. In panel (d) of Fig. 2 where $d=50, n=100,000$ there is close agreement between the empirical embedding probability and the Tracy–Widom limit.

**Fig. 3:**
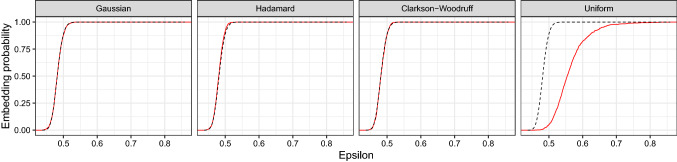
Analysis of subset of PKC$\varepsilon$ dataset $(n=407,779, d=132)$ with $B=1000$ sketches of size $k=20d$. The dashed black line and the solid red line gives the theoretical and empirical embedding probabilities respectively. The Tracy–Widom approximation is accurate for the Gaussian, Hadamard and Clarkson–Woodruff sketches. (Color figure online)

**Fig. 4:**
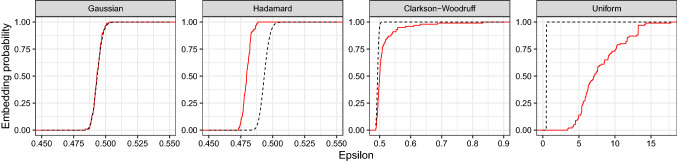
Analysis of full PKC$\varepsilon$ dataset $(n=407,779, d=1,034)$ with $B=100$ sketches of size $k=20d$. The *x*-axis is different in each panel.The dashed black line and the solid red line gives the theoretical and empirical embedding probabilities respectively. The Uniform projection is much less successful at generating $\epsilon$-subspace embeddings than the other data-oblivious projections. (Color figure online)

**Fig. 5:**
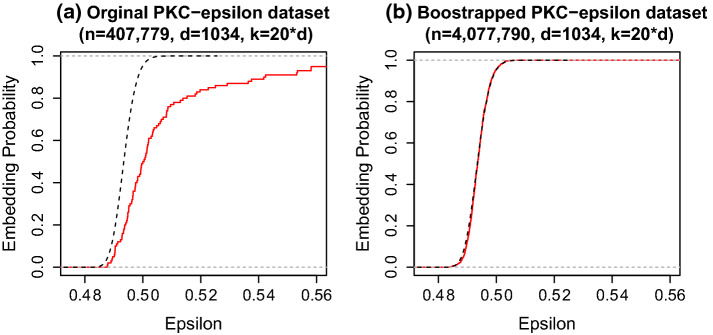
Comparison of results on the original PKC$\varepsilon$ dataset ($n=407,779$) and the bootstrapped larger PKC$\epsilon$ dataset ($n=4,077,790$). The dashed black line and the solid red line gives the theoretical and empirical embedding probabilities respectively. As expected from Theorem 3, the accuracy of the Tracy–Widom increases with *n*. (Color figure online)

## Data application

### $\epsilon$-subspace embedding

We tested the theory on a large genetic dataset of European ancestry participants in UK Biobank. The covariate data consists of genotypes at $p=1032$ genetic variants in the Protein Kinase C Epsilon (PKC$\varepsilon$) gene on $n=407,779$ subjects. Variants were filtered to have minor allele frequency of greater than one percent. The response variable was haemoglobin concentration adjusted for age, sex and technical covariates. The region was chosen as many associations with haemoglobin concentration were discovered in a genome-wide scan using univariable models; these associations were with variants with different allele frequencies, suggesting multiple distinct causal variants in the region. We also considered a subset of this dataset with $p=130$ representative markers identified by hierarchical clustering. When including the intercept and response, the PKC$\varepsilon$ subset has $n=407,779, d=132$, and the full PKC$\varepsilon$ dataset has $n=407,779, d=1034$.

The full PKC$\varepsilon$ dataset is of moderate size, so it was feasible to take the singular value decomposition of the full $n \times d$ dataset ${\varvec{A}}={\varvec{U}}{\varvec{D}}{\varvec{V}}^{\textsf{T}}$. Given the singular value decomposition we ran an oracle procedure to estimate the exact embedding probability. We generated *B* sketching matrices ${\varvec{S}}^{[1]}, \ldots , {\varvec{S}}^{[B]}$. These were used to compute $\epsilon ^{[b]} = \sigma _{\text {max}}({\varvec{I}}_{d}-{\varvec{U}}^{\textsf{T}}{\varvec{S}}^{[b]\textsf{T}}{\varvec{S}}^{[b]}{\varvec{U}})$ for $b=1, \ldots , B$ and give an estimated embedding probability as in (9). When working with the full PKC$\varepsilon$ dataset we simulated directly from the matrix normal distribution $\widetilde{{\varvec{U}}} \sim \text {MN}({\varvec{I}}_{k}, {\varvec{I}}_{d}/k)$ for the Gaussian sketch, rather than computing the matrix multiplication ${\varvec{S}}{\varvec{U}}$. We took $B=1000$ sketches of the PKC$\varepsilon$ subset, and $B=100$ sketches of the full PKC$\varepsilon$ dataset using the uniform, Gaussian, Hadamard and Clarkson–Woodruff projections, with $k=20\times d$.

Figure 3 shows the empirical and theoretical embedding probabilities for the PKC$\varepsilon$ subset $(n=407,779, d=132)$ for each type of sketch. The observed and theoretical curves match well for the Gaussian, Hadamard and Clarkson–Woodruff projection. The uniform projection performs worse than the other data-oblivious random projections, as larger values of $\epsilon$ indicate weaker approximation bounds. The uniform projection does not satisfy a central limit theorem for fixed *k*, so we do not necessarily expect the Tracy–Widom law to give a good approximation for the uniform projection.

Figure 4 shows the empirical and theoretical embedding probabilities for the full PKC$\varepsilon$ dataset $(n=407,779, d=1032)$ for each type of sketch. The Tracy–Widom approximation is accurate for the Gaussian sketch, but there are some deviations for the Hadamard and the Clarkson–Woodruff sketch. The empirical cdf for the Hadamard sketch (red) is to the left of the theoretical value (black), indicating smaller values of $\epsilon$ than predicted. This phenomenon is to be expected given the results on the extreme eigenvalues for the Hadamard sketch developed in Lacotte and Pilanci (2020). The distribution of $\epsilon$ has a longer right tail under the Clarkson–Woodruff sketch than is predicted by the Tracy–Widom law.

The deviation from the Tracy–Widom limit in Fig. 4 could be because the finite sample approximation is poor. Theorem 3 suggests that the Hadamard and Clarkson–Woodruff projections behave like the Gaussian sketch for *n* sufficiently large with respect to *d*. To test this we bootstrapped the full PKC$\varepsilon$ dataset to be ten times its original size. The bootstrapped PKC$\varepsilon$ dataset has $n=4,077,790, d=1034$. We took one thousand sketches of size $k=20\times d$ using the Clarkson–Woodruff projection and ran the oracle procedure of computing $\epsilon ^{[b]} = \sigma _{\text {max}}({\varvec{I}}_{d}-{\varvec{U}}^{\textsf{T}}{\varvec{S}}^{[b]\textsf{T}}{\varvec{S}}^{[b]}{\varvec{U}})$ for each sketch. Figure 5 compares the distribution of $\sigma _{\text {max}}({\varvec{I}}_{d}-{\varvec{U}}^{\textsf{T}}{\varvec{S}}^{\textsf{T}}{\varvec{S}}{\varvec{U}})$ using Clarkson–Woodruff projection on the original dataset and on the large bootstrapped dataset. As *n* increases we expect the quality of the Tracy–Widom approximation to improve. Panel (a) of Fig. 5 compares the theoretical to the simulation results on the original dataset. The Clarkson–Woodruff projection shows greater variance than expected. Panel (b) compares the theoretical to the simulation results on the bootstrapped dataset. In (b) there is very good agreement between the empirical distribution and the theoretical distribution. It seems that for this dataset $n \approx 400,000$ is not big enough for the large sample asymptotics to kick in. At $n \approx 4$ million the Tracy–Widom approximation is very good. As mentioned earlier, our motivation for using a sketching algorithm to perform data compression with tall datasets $n \gg d$. This example highlights that the asymptotic approximations become more accurate as the sample size *n* grows and the computational incentives for using sketching increase in parallel (Table 2).

**Table 2:** Mean sketching time (seconds) over ten sketches for each dataset

Projection	Subset $(p=132)$	Full $(p=1034)$
Gaussian	769	–
Hadamard	17.2	156
Clarkson–Woodruff	1.33	21
Uniform	0.03	2.8

The Gaussian sketch is considerably slower than the Hadamard and Clarkson–Woodruff sketches on the subset as is expected from Table 1

**Fig. 6:**
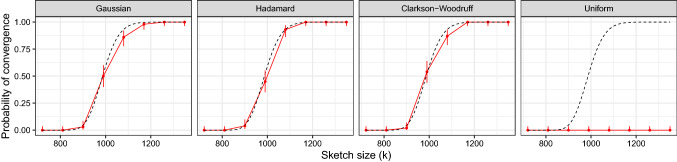
Convergence probability on year dataset $(n=515,344, d=91)$. Red solid points show the empirical convergence probability over $B=100$ sketches. The black dashed line gives the theoretical convergence probability using Theorem 2. The Tracy–Widom approximation is accurate for the Gaussian, Hadamard and Clarkson–Woodruff sketches. The uniform sketch fails to generate useful preconditioners. (Color figure online)

### Iterative optimisation

We considered iterative least-squares optimisation using the song year dataset available from the UCI machine learning repository. The dataset has $n=515,344$ observations, $p=90$ covariates, and year of song release as the response. We assessed the convergence probability by running the iteration (2) with the sketched preconditioner. The initial parameter estimate ${\varvec{\beta }}^{(0)}$ was a vector of zeros. The iteration was run for 2000 steps, with convergence being declared if the gradient norm condition $\Vert {\varvec{X}}^{\textsf{T}}({\varvec{y}}-{\varvec{X}}{\varvec{\beta }}^{(t)})\Vert _{2} < 10^{-6}$ was satisfied at any time step *t*. This convergence criterion was used instead of $\Vert {\varvec{\beta }}_{F} - {\varvec{\beta }}^{(t)} \Vert _{2}$ as ${\varvec{\beta }}_{F}$ will not be known in practice. This was repeated one hundred times for each of the random projections discussed in Sect. 2.1 using different sketch sizes *k*. Figure 6 compares the empirical (solid red points) and theoretical convergence probabilities (dashed black line) against the sketch size *k*. The point-ranges represent 95% confidence intervals. The Gaussian, Hadamard and Clarkson–Woodruff show near identical behavior, and the empirical convergence probabilities closely match the theoretical predictions using Theorem 2. The uniform sketch was much less successful in generating preconditioners, the algorithm did not show convergence in any replication at each sketch size *k*. In this example, the additional computational cost of the Gaussian, Hadamard and Clarkson–Woodruff sketches compared to the Uniform subsampling has clear benefits.

## Conclusion

The analysis of the asymptotic behavior of common data-oblivious random projections revealed an important connection to the Tracy–Widom law. The probability of attaining an $\epsilon$-subspace embedding (Definition 1) is an integral descriptive measure for many sketching algorithms. The asymptotic embedding probability can approximated using the Tracy–Widom law for the Gaussian, Hadamard and Clarkson–Woodruff sketches. The Tracy–Widom law can also be used to estimate the convergence probability for iterative schemes with a sketched preconditioner. We have tested the predictions empirically and seen close agreement. The majority of existing results for sketching algorithms have been established using non-asymptotic tools. Asymptotic results are a useful complement that can provide answers to important questions that are difficult to address concretely in a finite dimensional framework.

There was a stark contrast between the performance of the basic uniform projection and the other data-oblivious projections (Gaussian, Hadamard and Clarkson–Woddruff) in the data application. The Hadmard and Clarkson–Woodruff projections are expected to behave like the Gaussian projection under mild regularity conditions on the maximum leverage score. We observed this phenomenon when *n*/*d* was large, as is required by Theorem 3. The Hadamard and Clarkson–Woodruff projections are substantially more computationally efficient than the Gaussian projection (recall Table 1), so their universal limiting behavior implies that the trade-off between computation time and performance guarantees is asymptotically negligible in the regime (8).

The Tracy–Widom law has found many applications in high-dimensional statistics and probability (Edelman and Wang 2013), and we have shown that it useful for describing the asymptotic behavior of sketching algorithms. The asymptotic behaviour with respect to large *n* is of practical interest, as this is the regime where sketching is attractive as a data compression technique. The universal behavior of high-dimensional random matrices has practical and theoretical consequences for randomized algorithms that use linear dimension reduction (Dobriban and Liu 2018; Lacotte et al. 2020).

## Supplementary Information

Below is the link to the electronic supplementary material.

Supplementary file 1 (pdf 253 KB)
