# Supplementary material for: On randomized sketching algorithms and the Tracy–Widom law
Source: Stat Comput. 2023 Jan 19;33(1):34. doi: 10.1007/s11222-022-10148-5 (PMC9852177; doi:10.1007/s11222-022-10148-5)
Supplement: Supplementary file 1 — (pdf 253 KB) [file 11222_2022_10148_MOESM1_ESM.pdf]

Noname manuscript No.
(will be inserted by the editor)

Supplementary material for 'On randomized sketching algorithms and the Tracy-Widom law'

Daniel Ahfock · William J. Astle · Sylvia Richardson

Received: date / Accepted: date

1 Weak convergence

Our asymptotic arguments concern the convergence of sequences of probability measures. Billingsley (1999) is an authoritative reference on the topic. We now recap some useful foundational theory, as is presented in Van Der Vaart (1998, Chapter 2). The Portmanteau lemma gives a number of useful equivalent definitions of convergence in distribution (weak convergence).

Lemma S.1 (Portmanteau) *Let $(\boldsymbol{Z}_n)_{n\in\mathbb{N}}$ denote a sequence of random vectors of fixed dimension, and $\boldsymbol{Z}$ denote another random vector of the same dimension. The following statements are equivalent, where limits are being taken in n:*

(a) $\Pr(\boldsymbol{Z}_n \leq \boldsymbol{z}) \to \Pr(\boldsymbol{Z} \leq \boldsymbol{z})$ *at all continuity points $\boldsymbol{z}$ of the cumulative distribution function $\Pr(\boldsymbol{Z} \leq \boldsymbol{z})$.*
(b) $\Pr(\boldsymbol{Z}_n \in B) \to \Pr(\boldsymbol{Z} \in B)$ *for all Borel sets B with $\Pr(\boldsymbol{Z} \in \partial B) = 0$, where ∂B denotes the boundary of the set B. The boundary is defined as the closure of the set B minus the interior of B, so $\partial B = \overline{B} \setminus B^o$.*

Lemma S.2 (Uniform convergence) *Suppose that $(\boldsymbol{Z}_n)$ converges in distribution to a random vector $\boldsymbol{Z}$ with a continuous distribution function. Then*

$$\lim_{n\to\infty} \sup_{\boldsymbol{z}} |\Pr(\boldsymbol{Z}_n \leq \boldsymbol{z}) - \Pr(\boldsymbol{Z} \leq \boldsymbol{z})| = 0.$$

Proofs for these results are given in Chapter 2 of Van Der Vaart (1998).

2 Random Matrix Theory

Definition S.1 A random variable Z has a Tracy-Widom distribution F_1, when the cumulative distribution function is given by

$$F_1(z) = \exp\left(-\frac{1}{2}\int_z^{\infty} q(t) + (t-z)q^2(t)\ dt\right).$$

D. Ahfock, W.J Astle, S. Richardson
MRC Biostatistics Unit, University of Cambridge, Cambridge, UK.
E-mail: d.ahfock@uq.edu.au

Where $q(z)$ satisfies the nonlinear differential equation $q''(z) = zq(z)+2q^3(z)$, subject to the asymptotic boundary condition, $q(z) \sim \mathrm{Ai}(z) \quad \text{as } z \to \infty$. The function $\mathrm{Ai}(z)$ denotes the Airy function, defined as $\mathrm{Ai}(z) = \pi^{-1} \int_0^\infty \cos\left(t^3/3 + zt\right)\, dt$.

Theorem S.1 *(Ma, 2012)*
Consider a sequence of $\mathrm{Wishart}(k, \boldsymbol{I}_d/k)$ *random matrices where* $d, k \to \infty$ *and* $d/k \to \alpha$ *with* $\alpha \in (0, 1]$. *Let* $\lambda_{\max}$ *denote the maximum eigenvalue of the random matrix. Define the centering and scaling constants as*

$$\mu_{k,d} = k^{-1}(\sqrt{k-1/2} + \sqrt{d-1/2})^2, \quad \sigma_{k,d} = \frac{k^{-1}(\sqrt{k-1/2} + \sqrt{d-1/2})}{\left(1/\sqrt{k-1/2} + 1/\sqrt{d-1/2}\right)^{1/3}}.$$

Then with $Z \sim F_1$ *and* F_1 *is the Tracy-Widom distribution.*

$$\frac{(\lambda_{\max} - \mu_{k,d})}{\sigma_{k,d}} \xrightarrow{d} Z.$$

A limit theorem for the minimum eigenvalue is best expressed in terms of the logarithm of the minimum eigenvalue as this gives higher order accuracy (Ma, 2012).

Theorem S.2 *(Ma, 2012)*
Consider a sequence of $\mathrm{Wishart}(k, \boldsymbol{I}_d/k)$ *random matrices where* $d, k \to \infty$ *and* $d/k \to \alpha$ *with* $\alpha \in (0, 1]$. *Let* $\lambda_{\min}$ *denote the minimum eigenvalue of the random matrix. Set*

$$\begin{aligned} \mu_{k,d} &= (\sqrt{k-1/2} - \sqrt{d-1/2})^2, \\ \sigma_{k,d} &= (\sqrt{k-1/2} - \sqrt{d-1/2})\left(\frac{1}{\sqrt{k-1/2}} - \frac{1}{\sqrt{d-1/2}}\right)^{1/3}, \end{aligned}$$

and define the following centering and scaling constants $\tau_{k,d} = \sigma_{k,d}/\mu_{k,d}, \nu_{k,d} = \log(\mu_{k,d}) - \log k - \tau_{k,d}^2/8$. *Then where* $Z \sim F_1$ *and* F_1 *is the Tracy-Widom distribution,*

$$\frac{(\log \lambda_{\min} - \nu_{k,d})}{\tau_{k,d}} \xrightarrow{d} -Z,$$

3 Proof of Theorem 1

Proof The extreme eigenvalues of a Wishart random matrix converge in probability to fixed values as both the dimension and degrees of freedom expand. The result for the largest eigenvalue is due to Geman (1980) and the result for the smallest eigenvalue is due to Silverstein (1985).

Theorem S.3 *(Geman, 1980; Silverstein, 1985)*
Consider a sequence of $\mathrm{Wishart}(k, \boldsymbol{I}_d/k)$ *random matrices where the degrees of freedom* k *and dimension* d *are both taken to infinity. Suppose that the variables to samples ratio* d/k *converges to a constant* $(d/k) \to \alpha$, *where* $\alpha \in (0, 1]$. *Then the*

extreme eigenvalues of the random matrix, $\lambda_{\min}$ and $\lambda_{\max}$ converge in probability to the limits

$$(i)\ \lambda_{\min} \xrightarrow{p} (1-\sqrt{\alpha})^2, \tag{S.1}$$

$$(ii)\ \lambda_{\max} \xrightarrow{p} (1+\sqrt{\alpha})^2. \tag{S.2}$$

Theorem S.3 and the continuous mapping theorem can be used to determine the asymptotic embedding probability for the Gaussian sketch.

Lemma S.3 *Suppose we have an arbitrary $n \times d$ data matrix $\boldsymbol{A}_{(n)}$ where $n > d$ and $\boldsymbol{A}_{(n)}$ is of rank d. Assume we take a Gaussian sketch of size k. Then asymptotically in n, k and d, with $d/k \to \alpha$ where $\alpha \in (0, 1]$,*

$$\lim_{n,d,k\to\infty} \Pr(\boldsymbol{S} \text{ is an } \epsilon\text{-subspace embedding for } \boldsymbol{A}_{(n)}) = \begin{cases} 0 & \text{if } \epsilon < (1+\sqrt{\alpha})^2 - 1 \\ 1 & \text{if } \epsilon > (1+\sqrt{\alpha})^2 - 1 \end{cases}$$

Proof Let $\boldsymbol{W} \sim \text{Wishart}(k, \boldsymbol{I}_d/k)$, and let $\lambda_{\min}$ and $\lambda_{\max}$ denote the minimum and maximum eigenvalues of $\boldsymbol{W}$ respectively. Using Slutsky's theorem and the continuous mapping theorem we have the joint convergence result

$$\begin{bmatrix} |1-\lambda_{\min}| \\ |1-\lambda_{\max}| \end{bmatrix} \xrightarrow{p} \begin{bmatrix} |1-(1-\sqrt{\alpha})^2| \\ |1-(1+\sqrt{\alpha})^2| \end{bmatrix} = \begin{bmatrix} 2\sqrt{\alpha}-\alpha \\ 2\sqrt{\alpha}+\alpha \end{bmatrix}, \tag{S.3}$$

where the equality uses the fact that $\alpha \in (0, 1]$. For large k and d, the maximum eigenvalue $\lambda_{\max}$ is expected to show greater deviation from one than the minimum eigenvalue $\lambda_{\min}$. Over the interval $\alpha \in (0, 1]$ it holds that

$$|1-(1+\sqrt{\alpha})^2| > |1-(1-\sqrt{\alpha})^2|.$$

Applying the continuous mapping theorem to the random vector in (S.3),

$$\max \begin{bmatrix} |1-\lambda_{\min}| \\ |1-\lambda_{\max}| \end{bmatrix} \xrightarrow{p} \max \begin{bmatrix} |1-(1-\sqrt{\alpha})^2| \\ |1-(1+\sqrt{\alpha})^2| \end{bmatrix},$$

yields $\max(|1-\lambda_{\min}|, |1-\lambda_{\max}|) \xrightarrow{p} |1-(1+\sqrt{\alpha})^2|$. Now as $(1+\sqrt{\alpha})^2$ is greater than one for all $\alpha > 0$, the absolute value sign can be removed in the limit giving the equivalent statement $\max(|1-\lambda_{\min}|, |1-\lambda_{\max}|) \xrightarrow{p} (1+\sqrt{\alpha})^2 - 1$. Recalling that $\sigma_{\max}(\boldsymbol{I}_d - \boldsymbol{W}) = \max(|1-\lambda_{\min}|, |1-\lambda_{\max}|)$, we establish convergence of the limiting singular value

$$\sigma_{\max}(\boldsymbol{I}_d - \boldsymbol{W}) \xrightarrow{p} (1+\sqrt{\alpha})^2 - 1. \tag{S.4}$$

As convergence in probability to a constant implies convergence in distribution, the Portmanteau lemma then gives the probabilistic statement

$$\lim_{n,d,k\to\infty} \Pr(\sigma_{\max}(\boldsymbol{I}_d - \boldsymbol{W}) \leq \epsilon) = \begin{cases} 0 & \text{if } \epsilon < (1+\sqrt{\alpha})^2 - 1, \\ 1 & \text{if } \epsilon > (1+\sqrt{\alpha})^2 - 1. \end{cases}$$

As $\epsilon = (1+\sqrt{\alpha})^2 - 1$ is a discontinuity point of the limiting distribution function we do not make a statement about the case $\epsilon = (1+\sqrt{\alpha})^2 - 1$. We have the equality in limits

$$\lim_{n,d,k\to\infty} \Pr(\boldsymbol{S} \text{ is an } \epsilon\text{-subspace embedding for } \boldsymbol{A}) = \lim_{n,d,k\to\infty} \Pr(\sigma_{\max}(\boldsymbol{I}_d - \boldsymbol{W}) \leq \epsilon),$$

giving the final result.

Given Lemma S.3, we can move on to the proof of Theorem 1. Let $\boldsymbol{W} \sim \text{Wishart}(k, \boldsymbol{I}_d/k)$, and let $\lambda_{\min}$ and $\lambda_{\max}$ denote the minimum and maximum eigenvalues of $\boldsymbol{W}$ respectively. The majority of the proof comes down to showing that $\lambda_{\max}$ controls the embedding probability. Using the Portmanteau lemma (Lemma S.1) we will show that

$$\lim_{d,k\to\infty} \Pr(\sigma_{\max}(\boldsymbol{I}_d - \boldsymbol{W}) \leq \epsilon) = \lim_{d,k\to\infty} \Pr\left(|1-\lambda_{\max}| \leq \epsilon\right).$$

Recall the key expression

$$\begin{aligned}\Pr(\boldsymbol{S} \text{ is an } \epsilon\text{-subspace embedding for } \boldsymbol{A}) &= \Pr(\sigma_{\max}(\boldsymbol{I}_d - \boldsymbol{W}) \leq \epsilon) \\ &= \Pr\left(|1-\lambda_{\min}| \leq \epsilon, |1-\lambda_{\max}| \leq \epsilon\right).\end{aligned}$$

The Tracy-Widom law describes the marginal distributions of $\lambda_{\min}$ and $\lambda_{\max}$. We would like to avoid working with the joint distribution of the extreme eigenvalues, and instead restrict attention to the distribution of the maximum. Let $\boldsymbol{X}$ denote the random vector $\boldsymbol{X} = (|1-\lambda_{\min}|, |1-\lambda_{\max}|)^T$. Figure S1 presents some diagrams that will be useful. We wish to know the probability that $\boldsymbol{X}$ lies in the shaded region C in panel (a). For every $\epsilon > 0$ we have that $\Pr\left(|1-\lambda_{\min}| \leq \epsilon, |1-\lambda_{\max}| \leq \epsilon\right) = \Pr(\boldsymbol{X} \in C)$. The region C can be expressed as $C = M - R$ where M and R are the shaded regions in panels (b) and (c) respectively. The probability $\Pr(\boldsymbol{X} \in M)$ represents the marginal probability that $|1-\lambda_{\max}| \leq \epsilon$. The probability $\Pr(\boldsymbol{X} \in R)$ represents the probability of the joint event that $(|1-\lambda_{\max}| \leq \epsilon, |1-\lambda_{\min}| > \epsilon)$. We have that

$$\Pr(\boldsymbol{X} \in C) = \Pr(\boldsymbol{X} \in M) - \Pr(\boldsymbol{X} \in R).$$

In panel (c) the dot-dash line gives the identity line where $|1-\lambda_{\max}| = |1-\lambda_{\min}|$. From the first part of the proof of Lemma S.3 we know that as d, k tends to infinity $\boldsymbol{X}$ converges in distribution to the constant vector $\boldsymbol{X}_L = (|1-(1-\sqrt{\alpha})^2|, |1-(1+\sqrt{\alpha})^2|)^T$. As such, asymptotically $|1-\lambda_{\max}| > |1-\lambda_{\min}|$ with probability one. Referring to panel (c), the random vector $\boldsymbol{X}_L$ takes values in the region below the dot-dash line with probability one. The limiting random vector $\boldsymbol{X}_L$ thus satisfies $\Pr(\boldsymbol{X}_L \in R) = 0$ and $\Pr(\boldsymbol{X}_L \in \partial R) = 0$. As $\boldsymbol{X} \xrightarrow{d} \boldsymbol{X}_L$, Property (b) of the Portmanteau lemma (Lemma S.1) gives that $\Pr(\boldsymbol{X} \in R) \to \Pr(\boldsymbol{X}_L \in R) = 0$. The limiting probability is then

$$\begin{aligned}\lim_{d,k\to\infty} \Pr\left(|1-\lambda_{\min}| \leq \epsilon, |1-\lambda_{\max}| \leq \epsilon\right) &= \lim_{d,k\to\infty} \Pr(\boldsymbol{X} \in C) \\ &= \lim_{d,k\to\infty} \Pr(\boldsymbol{X} \in M) - \lim_{d,k\to\infty} \Pr(\boldsymbol{X} \in R) \\ &= \lim_{d,k\to\infty} \Pr(\boldsymbol{X} \in M) - 0 \\ &= \lim_{d,k\to\infty} \Pr\left(|1-\lambda_{\max}| \leq \epsilon\right). \qquad \text{(S.5)}\end{aligned}$$

We have now isolated the maximum eigenvalue $\lambda_{\max}$ as the determining factor in obtaining an ϵ-subspace embedding. We make another application of the Portmanteau lemma to arrive at the final result. From here we can write

$$\Pr\left(|1-\lambda_{\max}| \leq \epsilon\right) = \Pr(\lambda_{\max} \leq \epsilon + 1) - \Pr(\lambda_{\max} < 1 - \epsilon). \qquad \text{(S.6)}$$

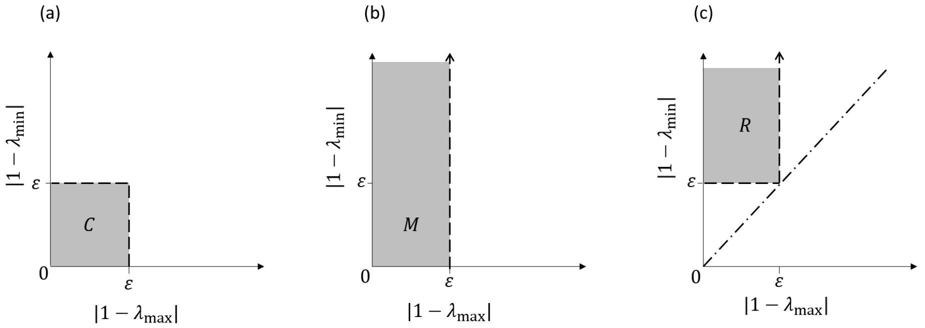

Fig. S1 Regions of interest in determining the embedding probability. To obtain an ϵ-subspace embedding we require that $|1-\lambda_{\min}| \leq \epsilon$ and $|1-\lambda_{\max}| \leq \epsilon$. If we define $\boldsymbol{X} = (|1-\lambda_{\min}|, |1-\lambda_{\max}|)^T$, we have that $\Pr(\boldsymbol{X} \in C) = \Pr(\boldsymbol{X} \in M) - \Pr(\boldsymbol{X} \in R)$. In panel (c) the dot-dash line gives the identity line where $|1-\lambda_{\max}| = |1-\lambda_{\min}|$.

From Theorem S.3 we know that $\lambda_{\max}$ converges in distribution to the constant random variable $Z_L = (1+\sqrt{\alpha})^2$, where we have assumed $\alpha \in (0,1]$. Let B denote the interval $(-\infty, 1]$. The limiting random variable Z_L satisfies $\Pr(Z_L \in B) = 0$ and $\Pr(Z_L \in \partial B) = 0$. As such using property b of the Portmanteau lemma, $\lim_{d,k\to\infty} \Pr(\lambda_{\max} \in B) = 0$. Now $\Pr(\lambda_{\max} \leq 1-\epsilon) \leq \Pr(\lambda_{\max} \in B)$ for any $\epsilon > 0$. We can then conclude that $\lim_{d,k\to\infty} \Pr(\lambda_{\max} \leq 1-\epsilon) = 0$ for any $\epsilon > 0$. Asymptotically, the term $\Pr(\lambda_{\max} \leq 1-\epsilon)$ drops out of the expression for the embedding probability. Taking limits over (S.6),

$$\begin{aligned}\lim_{d,k\to\infty} \Pr\left(|1-\lambda_{\max}| \leq \epsilon\right) &= \lim_{d,k\to\infty} \Pr(\lambda_{\max} \leq \epsilon+1) - \lim_{d,k\to\infty} \Pr(\lambda_{\max} \leq 1-\epsilon) \\ &= \lim_{d,k\to\infty} \Pr(\lambda_{\max} \leq \epsilon+1) - 0.\end{aligned}$$

The asymptotic embedding probability is then related to the asymptotic distribution of $\lambda_{\max}$. The inequality can be manipulated to include the centering and scaling constants that appear in Theorem S.1,

$$\lim_{d,k\to\infty} \Pr(\lambda_{\max} \leq \epsilon+1) = \lim_{d,k\to\infty} \Pr\left(\frac{\lambda_{\max}-\mu_{k,d}}{\sigma_{k,d}} \leq \frac{\epsilon+1-\mu_{k,d}}{\sigma_{k,d}}\right).$$

Let Z be a random variable with Tracy-Widom distribution F_1. For any fixed d and k, it must hold that for any fixed $\epsilon > 0$,

$$\begin{aligned}\left|\Pr\left(\frac{\lambda_{\max}-\mu_{k,d}}{\sigma_{k,d}} \leq \frac{\epsilon+1-\mu_{k,d}}{\sigma_{k,d}}\right) - \Pr\left(Z \leq \frac{\epsilon+1-\mu_{k,d}}{\sigma_{k,d}}\right)\right| \leq \\ \sup_{z\in\mathbb{R}}\left|\Pr\left(\frac{\lambda_{\max}-\mu_{k,d}}{\sigma_{k,d}} \leq z\right) - \Pr(Z \leq z)\right|.\end{aligned} \tag{S.7}$$

From Theorem S.1 we have that $(\lambda_{\max}-\mu_{k,d})/\sigma_{k,d}$ converges in distribution to the continuous random variable Z, where $Z \sim F_1$. It then follows from Lemma S.2 that

$$\lim_{d,k\to\infty} \sup_{z\in\mathbb{R}}\left|\Pr\left(\frac{\lambda_{\max}-\mu_{k,d}}{\sigma_{k,d}} \leq z\right) - \Pr(Z \leq z)\right| = 0.$$

Now by the squeeze theorem, it holds that for all $\epsilon > 0$,

$$\lim_{d,k\to\infty} \left| \Pr\left(\frac{\lambda_{\max} - \mu_{k,d}}{\sigma_{k,d}} \leq \frac{\epsilon + 1 - \mu_{k,d}}{\sigma_{k,d}} \right) - \Pr\left(Z \leq \frac{\epsilon + 1 - \mu_{k,d}}{\sigma_{k,d}} \right) \right| = 0.$$

From Theorem 1 of Ma (2012), the error in the approximation (S.7) is $O(d^{-2/3})$ for even d. As discussed in Ma (2012), it is difficult to give a rigorous error bound for odd d, however simulations suggest the $O(d^{-2/3})$ bound still holds.

4 Proof of Theorem 2

Proof Let $\boldsymbol{W} \sim \text{Wishart}(k, \boldsymbol{I}_d/k)$ and let $\lambda_{\min}$ denote the minimum eigenvalue of $\boldsymbol{W}$. The probability of convergence can be expressed as

$$\begin{aligned} \Pr\left(\lim_{t\to\infty} \|\boldsymbol{\beta}_F - \boldsymbol{\beta}^{(t)}\|_2 = 0\right) &= \Pr\left(\lambda_{\min} > 0.5\right) \\ &= \Pr\left(\frac{\log\lambda_{\min} - \nu_{k,d}}{\tau_{k,d}} > \frac{\log(0.5) - \nu_{k,d}}{\tau_{k,d}} \right) \quad \text{(S.8)} \end{aligned}$$

Let Z be a random variable with Tracy-Widom distribution F_1. Now from Theorem S.2, $(\log\lambda_{\min} - \nu_{k,d})/\tau_{k,d}$ converges in distribution to the continuous random variable $-Z$, where Z is distributed according to the Tracy-Widom distribution F_1. For any fixed d and k, it must hold that for any fixed $\epsilon > 0$,

$$\begin{aligned} &\left| \Pr\left(\frac{\log\lambda_{\min} - \nu_{k,d}}{\tau_{k,d}} > \frac{\log(0.5) - \nu_{k,d}}{\tau_{k,d}} \right) - \Pr\left(-Z > \frac{\log(0.5) - \nu_{k,d}}{\tau_{k,d}} \right) \right| \leq \quad \text{(S.9)} \\ &\qquad \sup_{z\in\mathbb{R}} \left| \Pr\left(\frac{\log\lambda_{\min} - \nu_{k,d}}{\tau_{k,d}} > z \right) - \Pr(-Z > z) \right|. \quad \text{(S.10)} \end{aligned}$$

From Lemma S.2 it must hold that

$$\lim_{d,k\to\infty} \sup_{z\in\mathbb{R}} \left| \Pr\left(\frac{\log\lambda_{\min} - \nu_{k,d}}{\tau_{k,d}} > \frac{\log(0.5) - \nu_{k,d}}{\tau_{k,d}} \right) - \Pr(-Z > z) \right| = 0.$$

Now by the squeeze theorem, for all $\epsilon > 0$,

$$\lim_{d,k\to\infty} \left| \Pr\left(\frac{\log\lambda_{\min} - \nu_{k,d}}{\tau_{k,d}} > \frac{\log(0.5) - \nu_{k,d}}{\tau_{k,d}} \right) - \Pr\left(-Z > \frac{\log(0.5) - \nu_{k,d}}{\tau_{k,d}} \right) \right| = 0.$$

Rearranging

$$\Pr\left(-Z > \frac{\log(0.5) - \nu_{k,d}}{\tau_{k,d}} \right) = \Pr\left(Z \leq \frac{\nu_{k,d} - \log(0.5)}{\tau_{k,d}} \right),$$

and using the identity (S.8) gives the the final result,

$$\lim_{n,d,k\to\infty} \left| \Pr\left(\lim_{t\to\infty} \|\boldsymbol{\beta}_F - \boldsymbol{\beta}^{(t)}\|_2 = 0 \right) - \Pr\left(Z \leq \frac{\nu_{k,d} - \log(1/2)}{\tau_{k,d}} \right) \right| = 0.$$

From Theorem 2 of Ma (2012), the error in the approximation (S.7) is $O(d^{-2/3})$ for even d. As discussed in Ma (2012), it is difficult to give a rigorous error bound for odd d, however simulations suggest the $O(d^{-2/3})$ bound still holds.

5 Proof of Theorem 3

Proof Assumption 1 on the leverage scores is sufficient to establish a central limit theorem for the data-oblivious sketches.

Theorem S.4 (Ahfock et al. (2020)) *Consider a sequence of arbitrary $n \times d$ data matrices $\boldsymbol{A}_{(n)}$, where d is fixed. Let $\boldsymbol{A}_{(n)} = \boldsymbol{U}_{(n)}\boldsymbol{D}_{(n)}\boldsymbol{V}_{(n)}^T$ represent the singular value decomposition of $\boldsymbol{A}_{(n)}$. Let $\boldsymbol{S}$ be a $k \times n$ Hadamard or Clarkson-Woodruff sketching matrix where k is also fixed. Suppose that Assumption 1 on the maximum leverage score is satisfied. Then as n tends to infinity*

$$[\widetilde{\boldsymbol{A}}\boldsymbol{V}_{(n)}\boldsymbol{D}_{(n)}^{-1} \mid \boldsymbol{A}_{(n)}] \xrightarrow{d} \mathrm{MN}(\boldsymbol{0}, \boldsymbol{I}_k, \boldsymbol{I}_d/k).$$

As we only need to consider the sequence of orthonormal matrices $\boldsymbol{U}_{(n)}$ to determine the embedding probability, we can use Theorem S.4 with $\boldsymbol{D}_{(n)}$ and $\boldsymbol{V}_{(n)}$ set to the $d \times d$ identity matrix. As such we conclude that $\boldsymbol{S}_{(n)}\boldsymbol{U}_{(n)} \xrightarrow{d} \mathrm{MN}(\boldsymbol{I}_k, \boldsymbol{I}_d/k)$. By the continuous mapping theorem it holds that for fixed d and k, asymptotically with n, $\boldsymbol{U}_{(n)}^T\boldsymbol{S}_{(n)}^T\boldsymbol{S}_{(n)}\boldsymbol{U}_{(n)} \xrightarrow{d} \mathrm{Wishart}(k, \boldsymbol{I}_d/k)$. Another application of the continuous mapping theorem gives

$$\sigma_{\max}(\boldsymbol{I}_d - \boldsymbol{U}_{(n)}^T\boldsymbol{S}_{(n)}^T\boldsymbol{S}_{(n)}\boldsymbol{U}_{(n)}) \xrightarrow{d} \sigma_{\max}(\boldsymbol{I}_d - \boldsymbol{W}),$$

where $\boldsymbol{W} \sim \mathrm{Wishart}(k, \boldsymbol{I}_d/k)$. We can use the continuous mapping theorem as the limiting Wishart matrix $\boldsymbol{W}$ has rank d with probability one. The maximum singular value function is continuous over the range where $\boldsymbol{W}$ has full rank (Bhatia, 1996). By the Portmanteau lemma it then holds that

$$\lim_{n\to\infty} \Pr\left(\sigma_{\max}(\boldsymbol{I}_d - \boldsymbol{U}_{(n)}^T\boldsymbol{S}_{(n)}^T\boldsymbol{S}_{(n)}\boldsymbol{U}_{(n)}) \leq \epsilon\right) = \Pr\left(\sigma_{\max}(\boldsymbol{I}_d - \boldsymbol{W}) \leq \epsilon\right).$$

Now as

$$\begin{aligned}\lim_{n\to\infty} \Pr\left(\boldsymbol{S}_{(n)} \text{ is an } \epsilon\text{-subspace embedding for } \boldsymbol{A}_{(n)}\right) &= \lim_{n\to\infty} \Pr\left(\sigma_{\max}(\boldsymbol{I}_d - \boldsymbol{U}_{(n)}^T\boldsymbol{S}_{(n)}^T\boldsymbol{S}_{(n)}\boldsymbol{U}_{(n)}) \leq \epsilon\right) \\ &= \Pr\left(\sigma_{\max}(\boldsymbol{I}_d - \boldsymbol{W}) \leq \epsilon\right),\end{aligned}$$

we have the final result.

6 Comment on Theorem 4

A random vector $\boldsymbol{Z} \in \mathbb{R}^d$ is said to be isotropic if $\mathbb{E}[\boldsymbol{Z}\boldsymbol{Z}^T] = \boldsymbol{I}_d$. Theorem 5.41 in Vershynin (2010) is reproduced below.

Theorem S.5 (Theorem 5.41 in Vershynin (2010)) *Let $\boldsymbol{A}$ be an $N \times n$ matrix whose rows $\boldsymbol{A}_i$ are independent isotropic random vectors in $\mathbb{R}^n$. Let m be a number such that $\|\boldsymbol{A}_i\|_2 \leq \sqrt{m}$ almost surely for all i. Then for every $t \geq 0$, one has*

$$\sqrt{N} - t\sqrt{m} \leq \sigma_{\min}(\boldsymbol{A}) \leq \sigma_{\max}(\boldsymbol{A}) \leq \sqrt{N} + t\sqrt{m}$$

with probability at least $1 - 2n \cdot \exp(-ct^2)$, where $c > 0$ is an absolute constant.

Suppose $\boldsymbol{S}$ is a uniform sketch of size k. As $\boldsymbol{U}^T\boldsymbol{U} = \boldsymbol{I}_d$, the matrix $\boldsymbol{B} = \sqrt{k}\boldsymbol{S}\boldsymbol{U}$ is a random $k \times d$ matrix whose rows are independent isotropic random vectors in $\mathbb{R}^d$. Now using the definition of the uniform sketch $\boldsymbol{B} = \sqrt{k}\boldsymbol{S}\boldsymbol{U} = \sqrt{k}\frac{\sqrt{n}}{\sqrt{k}}\boldsymbol{\Phi}\boldsymbol{U} = \sqrt{n}\boldsymbol{\Phi}\boldsymbol{U}$ where Φ subsamples k rows with replacement. Let $\boldsymbol{u}_i^T$ represent the i-th row in $\boldsymbol{U}$ and $\boldsymbol{b}_i^T$ represent the i-th row in $\boldsymbol{B}$. Now suppose that

$$\max_{i=1,\ldots,n} \|\boldsymbol{u}_i\|_2^2 \leq r.$$

Then the rows $\boldsymbol{b}_i^T$ of the matrix $\boldsymbol{B} = \sqrt{k}\boldsymbol{S}\boldsymbol{U} = \sqrt{n}\boldsymbol{\Phi}\boldsymbol{U}$ must satisfy

$$\max_{i=1,\ldots,k} \|\boldsymbol{b}_i\|_2^2 \leq \max_{i=1,\ldots,n} \|\sqrt{n}\boldsymbol{u}_i\|_2^2 = n \max_{i=1,\ldots,n} \|\boldsymbol{u}_i\|_2^2 \leq nr.$$

We can then apply Theorem 5.41 in Vershynin (2010) with $N = k, n = d$, $\boldsymbol{A} = \boldsymbol{B} = \sqrt{k}\boldsymbol{S}\boldsymbol{U}$, and $m = nr$. For every $t \geq 0$, with probability at least $1 - 2d\exp(-ct^2)$ one has

$$\sqrt{k} - t\sqrt{rn} \leq \sigma_{\min}(\sqrt{k}\boldsymbol{S}\boldsymbol{U}) \leq \sigma_{\max}(\sqrt{k}\boldsymbol{S}\boldsymbol{U}) \leq \sqrt{k} + t\sqrt{rn}.$$

Multiplication of each term by $1/\sqrt{k}$ then gives the bounds as stated in Theorem 4 of the main text,

$$1 - t\sqrt{\frac{rn}{k}} \leq \sigma_{\min}(\boldsymbol{S}\boldsymbol{U}) \leq \sigma_{\max}(\boldsymbol{S}\boldsymbol{U}) \leq 1 + t\sqrt{\frac{rn}{k}}.$$

References

Ahfock, D.C., Astle, W.J., Richardson, S.: Statistical properties of sketching algorithms. Biometrika **108**(2), 283–297 (2020)

Bhatia, R.: Matrix Analysis. Springer (1996)

Billingsley, P.: Convergence of Probability Measures. Wiley Series in Probability and Statistics. Wiley, New York, 2nd ed. (1999)

Geman, S.: A limit theorem for the norm of random matrices. The Annals of Probability **8**(2), 252–261 (1980)

Ma, Z.: Accuracy of the Tracy–Widom limits for the extreme eigenvalues in white Wishart matrices. Bernoulli **18**(1), 322–359 (2012)

Silverstein, J.W.: The smallest eigenvalue of a large dimensional wishart matrix. The Annals of Probability **13**(4), 1364–1368 (1985)

Van Der Vaart, A.: Asymptotic Statistics. Cambridge University Press (1998)

Vershynin, R.: Introduction to the non-asymptotic analysis of random matrices. arXiv preprint arXiv:1011.3027 (2010)
